# Pressure and Contact as Causes of Dental Decay

**Published:** 1876-08

**Authors:** Henry S. Chase

**Affiliations:** St. Louis.


### SELECTED ARTICLES.

### ARTICLE IV.

### *Pressure and Contact as Causes of Dental Decay.*

BY HENRY S. CHASE, M. D., D. D. S., ST. LOUIS.

From observations continued during the practice of my profession for the last thirty-one years, I must say that the consideration of the two factors, contact and pressure, demands an amount of attention surpassed only by that which takes cognizance of those causes which produce imperfect genesis and development of the dental organs.

In what I now have to say, I am aware that I shall state only that which I have advanced on former occasions; but as "line upon line and precept upon precept" seems to be as necessary in modern times as in the days of the ancient philosophers, I am content to do so. And if I dogmatize, it is only the result of convictions founded on my own experience and observations.

We do not live in a state of nature. That which we call a natural condition is only the result of the long struggle of man with his adverse surroundings and an effort to adapt himself to the conditions in which he finds himself, with those adaptations or modifications of organism impressed on thousands of generations and intensified by inheritance. For, by observing the state of the physical organism in the savage man of the near-past generations, we find that the teeth are in contact without lateral pressure.

Therefore we may say that it is the *natural* condition of the teeth to be in mutual contact. In the savage state the teeth do not decay, as a rule. The mode of life is such that the teeth have been well generated and developed. Furthermore, the diet of primitive man was such as to cause no acid formations in the mouth which might prove destructive to the teeth. Thus, in this primitive or natural condition, there was no dental decay, notwithstanding contact. Contact, alone, is not a cause of decay ; but contact combined with unfavorable physiological and chemical conditions, is a great source of dental destruction.

Modern man has teeth inferior in both anatomical and physiological structure to primitive man. Fissures of the enamel do not belong to the perfect development of the teeth found in uncivilized nations. A more perfect condition of calcification is also found in those teeth than in those of modern civilized nations, as is seen in the greater specific gravity of the former.

The genesis and growth of the teeth, together with the diet of primitive man, being in perfect harmony, the result of mutual adaptation and heritage for untold ages rendered contact innocuous. Modern or civilized man has the reverse of these conditions, and the results are decay and disease.

*Pressure.*—Primitive man and savage nations of the near-past were exclusive in regard to their intercourse with other tribes or nations.

They neither married or gave in marriage to other tribes except on rare occasions.

As a consequence, the teeth were in perfect harmony with the jaws and each other, as to topographical relations. That harmony had become fixed by in-and-in breeding.

The type, by inheritance, became confirmed. Civilized man, by his promiscuous intercourse, has invited outside marriages ; and by cross-breeding, without regard to the *law* of breeding, the teeth of *modern* Americans, especially are not in harmonious topographical relationship.

By the extraordinary influx of all nations into these States, the immigrants of Ireland and Germany especially, the teeth of Americans to-day are the resultant of the intermarriages of these foreign people with those of the old Anglo-American stock, giving us at this time millions of dental arches crowded and decaying.

There has always been an excess of foreign males over females. A much larger proportion of foreign males have married American females than have American males married foreign females.

The Germans have larger teeth than the Anglo-American. The teeth of the progeny of a German male and an Anglo American female seem to be too large for their maxillæ. The German male gives the locomotive system and the skin, hair, and teeth, while the Anglo-American female gives the nutritive system and maxillæ.

At first glance it would seem that the teeth belong to the nutritive system ; but it must be remembered that they belong to the dermoid system, being developed from that tissue.

Those who would like to study the laws of breeding would do well to read " Walker on Intermarriage".

I practiced dentistry in Woodstock, Vermont, from 1844 to 1857. The population of the country was entirely native and agricultural. The inhabitants were all descendants of the first settlers of Connecticut or Massachusetts. They were a distinct and homogeneous race, having a fixed anatomical type. The size of the teeth was in harmonious relation with the jaws in which they were grown. In my practice in the town and country I never saw a case of irregularity which demanded the least treatment.

At the present day, in nearly all parts of this country, the population is a mixed one, and cross-marriages have taken place to a great extent, so that there is no fixed anatomical type; and it will require several generations before this result is attained.

We find, at this day, large numbers of children with irregular and crowded dentures; and in adult life, if they are not then very irregular, they are still crowded to such an extent that it is with the greatest difficulty the third molars are erupted, many of them taking abnormal positions, or not appearing at all through the gums. The consequence is undue lateral pressure of nearly all the teeth on their proximate surfaces.

*Contact* results in decay from chemical disintegration of food left between the teeth, thus producing acids which act as solvents to them. This may be obviated to a considerable extent by great attention to cleansing the surfaces in contact; but in the greater number of individuals this hygienic measure is not practiced. In cases of pressure the same chemical results follow as in contact. But those hygienic measures which would operate favorably in contact will not produce the desired end in pressure; for in the latter there is caused a physiological change in the enamel by obstruction of the circulation of its nutritive fluids, producing at last a necrosis of that part of the enamel subjected to the pressure. I hope it is unnecessary, in presence of this learned body, to defend the statement that the enamel, as well as the dentine and cementum, has a circulation of blood plasma, giving it vital relations with other portions of the tooth.

My study and observations have taught me that necrosis of the enamel takes place from abnormal pressure. This is more evident in the teeth of young persons than in those of mature age. After enamel necrosis has taken place from pressure, chemical forces play their part with far more effect than when that tissue remains alive.

For, beside the enamel necrosis, and before chemical disintegration of that part takes places at all, in a large number of instances I have found a decalcification of that portion of dentine immediately in connection with the enamel, evidently the result of irritation produced by the decomposition of blood plasma in the dead and dying enamel.

One will often find the surface of the necrosed enamel *intact*, hard, and glassy, and of a color varying from brown to black; but on cutting into it there will be observed only a very thin layer that is hard and glassy. Immediately it will be found that it is softer, brittle, and chalk-like. The farther it is penetrated the lighter becomes the color. When the dentine is reached, that tissue will be found more or less decalcified, but of a lighter color than the enamel. After chemical disintegration of the latter takes place, the decalcified dentine succumbs so rapidly to decay that a comparatively large cavity is immediately produced.

What is the remedy? "Prevention is better than cure." Better, because, when applied to the teeth, a cure is not a perfect one. Certainly it is better to have *whole* teeth than those which have been decayed and plugged, even if the operation has been as perfectly performed, in every instance, as the present condition of dental art will allow.

Certainly it is better to have whole and sound teeth, than those which have been filled and cut away on their proximate surfaces. The latter process I shall call section.

None will deny the value of plugging. Many persons deny the value of section. I am not one of the latter. But I am in favor of reducing the number of teeth which shall demand either plugging or section. How can this be done? By reducing the number of teeth in the mouth, thereby giving plenty of room to those which remain.

Extraction, then, is the remedy! The question immediately arises, what teeth shall be extracted, and at what age shall the operation be performed? The practice must vary according to circumstances. I think those teeth which are the most liable to decay, and also the most difficult to preserve permanently by plugging, posterior to the canines. My experience unhesitatingly points to the bicuspids and first molars. In some cases the extraction of one bicuspid on each side and in either jaw will be sufficient to give the required room. In other cases the loss of both a bicuspid and a molar will be necessary. The mouth looks much better with a bicuspid in near relation to the canine than with a molar in that position, as the transition in size is not so apparent in the former as in the latter case. The fact of the second bicuspid being more liable to decay than the first would indicate its extraction, generally; though in the exceptions which may be found the reverse treatment would be the proper one.

The eruption of the first molar being so many years in advance of the bicuspids, our attention will naturally be called to the condition of this tooth. When it requires plugging before the age of eleven years, I think it should at that age be extracted, if more room than the diameter of a bicuspid is desired. And if the first molar is one which would not probably be retained, even by plugging, at least twenty or thirty years, I would advocate its extraction, even if the prospect should be the ultimate extraction of a bicuspid at the age of thirteen or fourteen years. If the sixth year molar is extracted at the age of eleven or twelve, the second molar will *not* tilt forward, but will, in most cases, retain an upright, natural position, and move bodily forward to some extent toward the open space. If this molar is extracted at the age of seven or eight years, the second molar will nearly always occupy the most of the room formerly held by the first.

The proper age at which to remove the bicuspids, it seems to me, is at the age of thirteen or fourteen, at least, after the full eruption of the second molar,—that we may be able to judge *which* of the bicuspids ought to be extracted.

Without doubt the majority of people value the canines and incisors above all the other teeth. Their perfection is greatly desired. The condition of the six anterior teeth, then, regarding pressure or caries, should lead the dentist to decide whether to extract the first or the second bicuspid. I have often found that the removal of the latter would relieve the condition of pressure by the movement of the first bicuspid posteriorly into the second bicuspid space.

*The Wisdom Teeth.*—I have frequently observed that bicuspids which have been only in contact previous to the development and eruption of the third molars have afterward suffered from pressure caused by the forward movement of the first and second molars. Even the incisors feel the effects of this forward movement, the effect of the *late comers.*

The wisdom-teeth are erupted after mal-condition of the previously-formed teeth may have taken place. Poor as their organization generally is, we cannot choose between *their* extraction and that of the first molars, for the purpose under consideration, owing to the fact of their later eruption. But defective as we find them, they are of easier preservation than a decayed first molar or bicuspid, provided the latter is diseased on the proximate surfaces.

If these wisdom-teeth receive proper professional attention immediately after eruption, they are usually as lasting as any of the other molars. I have often observed them in mouths where the other molars and bicuspids had all been lost by decay.

*General Observations*—I do not regard the violation of the laws of cross breeding as the only cause of irregularity or pressure. Undoubtedly there are other causes. Among these I would suggest the rapid intellectual development of the race, causing a retrograde evolution in regard to the size of both jaws.

Is not the vital system, as a whole, undergoing the same change? Are not the changes which have taken place in the food of civilized man among the causes for a diminished vital system? Has not the change from the diet of the savage and hunter to that of the modern confectioner produced a smaller jaw.

When I speak of *circulation* in the enamel, I mean a movement of the nutritious portion of the blood through the animal basis of the enamel fibres to that extent which will give it regular nourishment. It matters not whether that movement be a rapid or a sluggish one, or whether the fluid circulating in the enamel is changed hourly, weekly, or yearly. It is enough that there *is* a circulation, and that nourishment of tissue is secured by its means.

It must not be supposed that I advocate the universal practice of extraction, which I have detailed. I expect men will use "common sense" in this, as well as in other matters of professional judgment. At the age at which I have recommended the operation of extraction, it must be observed that the full growth of the jaws is not attained. By seeing both the father and the mother of the child, it may be noted which has given the locomotive, and which the nutritive, system. If the father has given the locomotive system, then it may be judged with certainty that the mother has given the nutritive or vital system. If she has given the nutritive system, then she has, with great probability, given the maxillæ. The size, then, of the maxillæ of the now child, at adult life, may be determined by those possessed by the mother; modified, it may be, to a slight extent, by larger or smaller locomotive system of the father The same principles apply when the father has given the nutritive system and the mother the locomotive system.

As before remarked, man on this continent is in a transitive state. It will be a long time before the race here will become homogeneous.

It is during this transition period that I think the practice which I recommend will be advantageous.

It is probable that no one can tell what the future anatomical condition of the maxillæ will be.

That they might be shortened after the lapse of many generations, by constant extraction of the teeth at an early age, and by "in and in" breeding, I do not doubt; but long before that the race will have become homogeneous, and harmony will prevail between the teeth and the jaws, if no other disturbing cause interfere.

One disturbing element I have already hinted at, namely, the *intellectual* growth of man to the exclusion of his *physical* development.

With the increase in the capacity of the brain-case there seems to be a decrease in the size of the maxillæ.

The substitution of food which requires but little exercise and power of the masticatory muscles will, in a long series of generations, at least diminish the size of the jaws.

This evolution of food is now going on with regard to the *Race*, without regard to the intellectual development of the *Individual.—Missouri Dental Journal.*

